# Tumor Suppressive Effects of miR-124 and Its Function in Neuronal Development

**DOI:** 10.3390/ijms22115919

**Published:** 2021-05-31

**Authors:** Rikako Sanuki, Tomonori Yamamura

**Affiliations:** Department of Applied Biology, Kyoto Institute of Technology, Saga Ippongi-cho 1, Ukyo-ku, Kyoto 616-8354, Japan; b7111050@edu.kit.ac.jp

**Keywords:** microRNA-124, tumor suppression, EMT, metastasis, neuronal development

## Abstract

MicroRNA-124 (miR-124) is strongly expressed in neurons, and its expression increases as neurons mature. Through DNA methylation in the miR-124 promoter region and adsorption of miR-124 by non-coding RNAs, miR-124 expression is known to be reduced in many cancer cells, especially with high malignancy. Recently, numerous studies have focused on miR-124 due to its promising tumor-suppressive effects; however, the overview of their results is unclear. We surveyed the tumor-suppressive effect of miR-124 in glial cell lineage cancers, which are the most frequently reported cancer types involving miR-124, and in lung, colon, liver, stomach, and breast cancers, which are the top five causes of cancer death. Reportedly, miR-124 not only inhibits proliferation and accelerates apoptosis, but also comprehensively suppresses tumor malignant transformation. Moreover, we found that miR-124 exerts its anti-tumor effects by regulating a wide range of target genes, most notably *STAT3* and *EZH2*. In addition, when compared to the original role of miR-124 in neuronal development, we found that the miR-124 target genes that contribute to neuronal maturation share similarities with genes that cause cancer cell metastasis and epithelial-mesenchymal transition. We believe that the two apparently unrelated fields, cancer and neuronal development, can bring new discoveries to each other through the study of miR-124.

## 1. Introduction

MicroRNAs (miRNAs) are short, single-stranded RNAs consisting of approximately 20 nucleotides. The first miRNA discovered was the *lin-4* in *Caenorhabditis elegans* [1,2]. Since then, many miRNAs have been identified in many species. miRNAs play a role in regulating post-transcriptional gene expression by forming an RNA-inducible silencing complex, which binds complementarily to the 3′UTR of mRNAs and cleaves or represses translation of the mRNA. Genes whose expression is regulated by miRNAs are called target genes, and a single miRNA could potentially regulate thousands of target genes. Therefore, miRNAs contribute to a wide range of processes, from morphogenesis to disease development, carcinogenesis, and its progression. miRNAs that promote cancer development are called oncomirs, while miRNAs that suppress cancer development are called tumor suppressive miRNAs (ts-miRNAs). Although many ts-miRNAs exist [3,4], we are focusing on one microRNA, microRNA-124 (miR-124), which is important in neurogenesis. Elsewhere, epigenetic changes of miRNAs in cancer cells can also be useful as biomarkers; however, this is outside the scope of this text and is summarized in other literatures [5,6,7].

miR-124 is derived from three independent genes (*miR-124-1*, *miR-124-2*, and *miR-124-3*), and is one of the most highly expressed miRNAs in the central nervous system [8]. It is particularly expressed in neurons and is involved in their maturation and function [9,10]. Aside from being extremely abundant in neurons, miR-124 is thought to also play an important role in suppressing the oncogenic transformation of normal cells in other tissues, even if the miR-124 expression level is considerably lower than that in neurons. In recent years, miR-124 has been increasingly reported as an miRNA with antitumor activity. However, many reports with unclear overview of their results exist. Therefore, in this review, we summarize the anti-tumor effects of miR-124. We then discuss the functions of miR-124 with that of its original role in neurons.

## 2. Suppression of miR-124 Functions in Cancer Cells

miRNAs are downregulated globally in human cancers, suggesting their role as general tumor suppressors [11]. Among them, the expression level of miR-124 is decreased in cancer cells compared to that in normal tissues, and is presumably involved in the pathogenesis of cancer. In 2007, the decreased expression of miR-124 was discovered to be caused by methylation of the CpG islands of the genomic region encoding miR-124 in colorectal cancer cells HCT-116 [12]. This finding led to the further discovery of the tumor-suppressive effect of miR-124. Genes whose expression is regulated by miRNAs are called target genes. The *CDK6* gene in HCT-116 cells, which is involved in cell cycle progression, was identified as a target gene of miR-124 [12,13]. Subsequently, miR-124 expression was also found to be reduced in glioblastoma multiforme and medulloblastoma compared to normal tissues, and exogenous miR-124 expression was found to be effective in inhibiting cancer cell growth by targeting *CDK6* [14,15].

The suppression of miR-124 functions in cancer cells is not limited to transcriptional regulation. Some kinds of protein non-coding RNAs have sequences complementary to miR-124 and are thought to suppress the function of miR-124 in cancer cells by adsorbing miR-124. Circular RNAs (circRNAs) have been reported to regulate cell growth by sponging multiple miRNAs, including miR-124 [16,17,18,19,20,21,22,23]. Reportedly, the circRNA, circHIPK3, which was derived from Exon2 of *HIPK3* [16], contributes to cancer progression by adsorbing miR-124 in many types of cancer, including liver cancer [16,24], glioma [25,26,27], lung cancer [28], gastric cancer [29], gallbladder cancer [30], and oral squamous cell carcinoma [31]. In addition to circRNAs, the long non-coding RNA, metastasis-associated lung adenocarcinoma transcript-1 (MALAT1), can also adsorb miR-124 and promote non-small cell lung cancer [32,33], cervical cancer [34], and nasopharyngeal carcinoma [35], and is known to promote malignancy through increased expression of miR-124 target genes.

**Figure 1:**
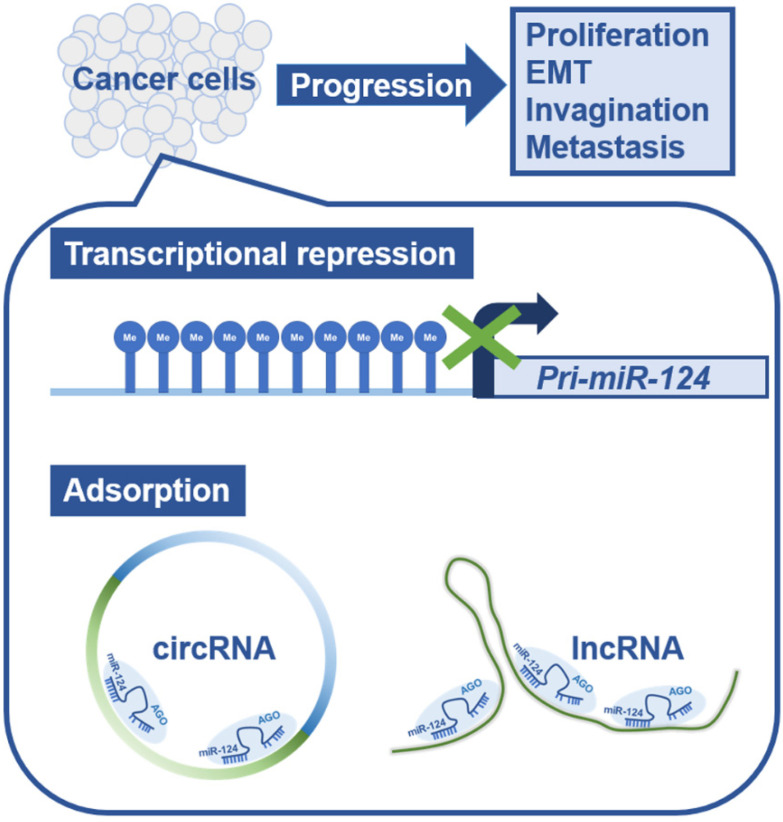
Schematic diagram of the mechanism of microRNA-124 functional repression in cancer cells. In human cancer cells, miRNA expression is generally downregulated, triggering the progression of cancer pathology. miR-124 transcription is repressed by methylation of CpG islands in the genomic region encoding pri-miR-124. In addition, miR-124 is adsorbed and removed by circular RNAs and long non-coding RNAs that have sequences complementary to miR-124. Abbreviations: pri-miR-124, primary microRNA-124; circRNA, circular RNA; lncRNA, long non-coding RNA; EMT, epithelial-mesenchymal transition; AGO, Argonaute.

Thus, a great variety of cancers create a more favorable environment for tumor growth either by suppressing miR-124 expression, by adsorbing miR-124, or in combination (Figure 1).

## 3. Tumor Suppressive Effects by miR-124 in Glial Lineage Cancers

Although miR-124 is downregulated in various types of cancers and exhibits tumor-suppressive effects through overexpression, the most frequently reported cancers associated with miR-124 are those of the glial cell lineage, such as glioblastomas and astrocytomas [15,36,37,38,39,40,41,42,43,44,45,46,47,48,49,50]. Reportedly, expression of the target gene of miR-124 is consistent with malignancy and prognosis [38,41,51]. Many miR-124 target genes have been identified, including CDK6, and miR-124 can not only suppress cell proliferation and progress apoptosis but also inhibits cell invasion, metastasis, and angiogenesis by reducing the expression of the target genes. Thus miR-124 is expected to be effective in almost all processes of anti-tumor effects including tumor malignant transformation (Table 1). Moreover, interestingly, it has been reported that miR-124 expression affects not only the glioma stem cells but also the cancer-immune system by activating T cells through the glioma stem cells [39]. Temozolomide (TMZ) is an anticancer drug that damages DNA by methylating guanine, induces cell cycle arrest, and causes cell death due to cellular stress [52]. TMZ is the first-line chemotherapeutic agent in glioblastoma, and its effect is reportedly reinforced by miR-124 [45,48]. Therefore, the combination of miR-124 with anticancer drugs is expected to be more effective in glioma chemotherapy.

## 4. Tumor Suppressive Effects of miR-124 in Major Cancers

According to a WHO study in 2020, lung, colorectal, liver, gastric, and breast cancers account for half of all worldwide cancer deaths. Many studies and reports exist that prove miR-124 is effective against all five of these cancers.

In the pathogenesis of lung cancer, Kirsten rat sarcoma viral oncogene homologue (*KRAS*) mutation-driven lung cancer causes increased aggressiveness and tumor size by gene ablation of the miRNA processing enzyme *DICER1* [53], suggesting that miRNA functions are important for suppressing cancer. In non-small cell lung cancer (NSCLC), miR-124 suppresses cell proliferation, inhibits invasion and metastasis, and induces apoptosis [32,54,55,56,57,58,59,60,61,62,63,64,65,66,67,68] (Table 2).

Epithelial-mesenchymal transition (EMT) is a differentiation mechanism that results in the acquisition of undifferentiated traits during carcinogenesis. Reportedly, miR-124 suppresses EMT in NSCLC cells. According to these studies, miR-124 suppresses enhancer of zeste homolog 2 (*EZH2*) and zinc finger E-box binding homeobox 1 (*ZEB1*) [57,67], which are transcription factors that promote EMT, and also targets N-cadherin (*CDH2*) [68]. Furthermore, in cells with *KRAS* mutations that cause EMT, miR-124 leads to cell death by suppressing autophagy, which is not observed in cancer cells with wild-type *KRAS* [59]. The same miR-124 target genes may or may not be targeted by miR-124 depending on the presence of *KRAS* mutation, which is interesting from the viewpoint of the gene targeting mechanism of miRNAs.

In colorectal cancer, miR-124 inhibits tumor formation by suppressing the proliferation of cancer cells and inhibiting metastasis [69,70,71]. Additionally, miR-124 expression increases oxidative stress and induces apoptosis [72]. Malignant tumors are known to have a glycolytic bias in glucose metabolism, known as the Warburg effect, which is thought to result from the adaptation of malignant tumors to a hypoxic environment [73]. miR-124 has also been reported to suppress the Warburg effect in a study on colorectal cancer [74] (Table 3).

In liver cancer, miR-124 is expected to have tumor-suppressive effects, such as arrest of cell proliferation, induction of apoptosis, and inhibition of invasion and metastasis by inhibiting EMT [77,78,79,80,81,82,83] (Table 4). The chloride intracellular channel 1 (*CLIC1*) is a chloride intracellular channel, and its expression is upregulated in many cancer cells. Many studies have focused on changes in cytoskeleton-related genes in the suppression of EMT, but in liver cancer, a channel called *CLIC* has been identified as a target of miR-124 and is effective in suppressing metastasis and invasion [83].

It is known that miR-124 is also downregulated in gastric cancer cell lines, and the expression of miR-124 has been reported to inhibit growth, decrease colony-forming ability, induce apoptosis, and suppress metastasis and invasion [84,85,86,87,88] (Table 5). In gastric cancer cells, the transfection of miR-124 can reportedly enhance the anticancer effect of 5-fluorouracil [88].

miR-124 is also known to be downregulated in breast cancer cells and has been reported to have anticancer activity [89,90,91,92,93,94,95,96,97,98] (Table 6). Although apoptosis is not induced in breast cancer [90], cell cycle arrest and inhibition of invasion and metastasis are the major tumor suppressive effects of miR-124. In breast cancer cells, miR-124 expression can suppress metastasis by targeting and regulating genes that contribute to cytoskeletal dynamics, such as connective tissue growth factor (*CTGF*), ras homolog family member G (*RHOG*), integrin beta-1 (*ITGB1*), and rho-associated coiled-coil-containing protein kinase (*ROCK1*) [89]. Bone is a highly favorable environment for the colonization and growth of metastatic tumors, and breast cancer patients are particularly prone to skeletal metastasis. Osteolysis is triggered by cancer cells to invade the bone [99]. Reportedly, miR-124 also inhibits bone metastasis by suppressing interleukin 11 (*IL11*) expression in breast cancer cells, regulating osteoclastogenesis, and reducing osteolysis [96]. The mechanism of inhibiting cancer progression by regulating the differentiation of other cells through the target gene represents the diverse anticancer effects of miR-124.

**Figure 2:**
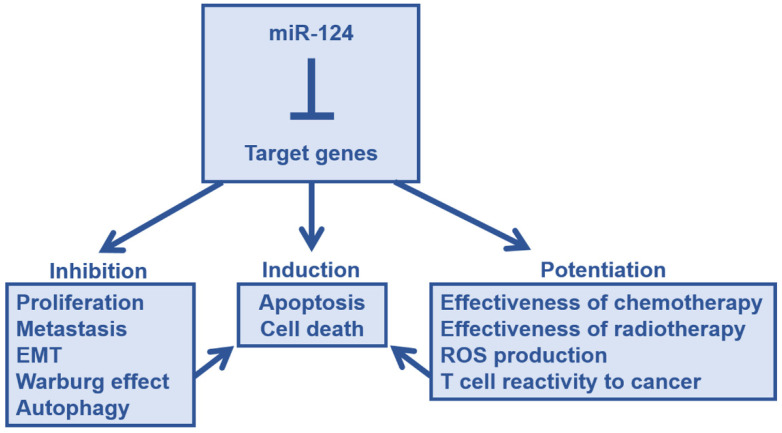
The antitumor effect of microRNA-124. Through the suppression of target gene expression, miR-124 prevents cancer progression and enhances therapeutic effects. These factors collectively lead to direct tumor suppression effects, such as cancer cell death. Abbreviations: ROS, reactive oxygen species; EMT, epithelial-mesenchymal transition.

Thus, miR-124 has tumor-suppressive effects on various tumors, such as inhibition of cell growth, invagination, migration, metastasis, invasion, and EMT. Moreover, miR-124 also alters the metabolism of cancer cells, thereby suppressing the Warburg effect. It also causes a decrease in autophagy function. In addition, miR-124 expression enhances the efficacy of established therapies, such as improving the sensitivity of treatment-resistant cancer cells (Figure 2). We believe that the effect of miR-124 will have a positive impact on cancer immunotherapy as well, spreading to the cells surrounding the cancer cells expressing miR-124.

## 5. Target Genes Responsible for the Tumor Suppressor Effect of miR-124, *STAT3*, and *EZH2*

The detailed mechanism of gene targeting by miRNAs remains unclear, and the same target gene may be targeted, or not targeted, in different cell types [59]. Therefore, we examined the target genes of miR-124 commonly responsible for tumor suppression in many cancer cells.

Among the many target genes of miR-124, signal transducer and activator of transcription 3 (*STAT3*) is the most popular gene shared by many types of cancer cells and is reportedly a target gene in glioma [39], lung cancer [32,55,63,65], colorectal cancer [75,76], hepatocellular carcinoma [80], breast cancer [97,100], endometrial cancer [101], esophageal cancer [102,103], nasopharyngeal carcinoma [104], retinoblastoma [105], prostate cancer [106], and cholangiocarcinoma [107]. In gastric cancer, miR-124 and *STAT3* have already been used to evaluate drug efficacy in anticancer activity [108]. STAT3 is a transcription factor that is activated by phosphorylation which in turn activates the expression of anti-apoptosis-related genes [109]. Moreover, malignant transformation of cells is mediated by the activation of STAT3, and targeting STAT3-signaling reduces the susceptibility of many cell types to malignant transformation [110,111]. Therefore, the fact that miR-124 targets *STAT3* is an excellent explanation for the molecular mechanism of the anti-tumor effect of miR-124. Note that STAT3 suppresses apoptosis, but can also promote apoptosis [112,113], which may be the cause of the difference in the induction of apoptosis by miR-124 in different cancer cell types and environments.

Another frequently reported target gene of miR-124 is the enhancer of zeste homolog 2 (*EZH2*), which has been found in lung adenocarcinoma [67], hepatocellular carcinoma cancer [87,88], cholangiocarcinoma [107], laryngeal squamous cell carcinoma [114], multiple myeloma [115], and ovarian cancer [23]. *EZH2* is reportedly essential for the proliferation of cancer cell lines and for regulating the expression of genes related to EMT [116,117]. Therefore, it is responsible for miR-124-induced tumor suppression in cell growth inhibition [50,67,87] and EMT inhibition [23,67,79,88]. In cholangiocarcinoma, miR-124 targets both *EZH2* and *STAT3*, and more interestingly, knockdown of *EZH2* is associated with a decreased expression of *STAT3*. It has also been reported that *EZH2-STAT3* causes autophagy-related death [107].

## 6. Comparison with Neuronal Development, the Original Function of miR-124

**Figure 3:**
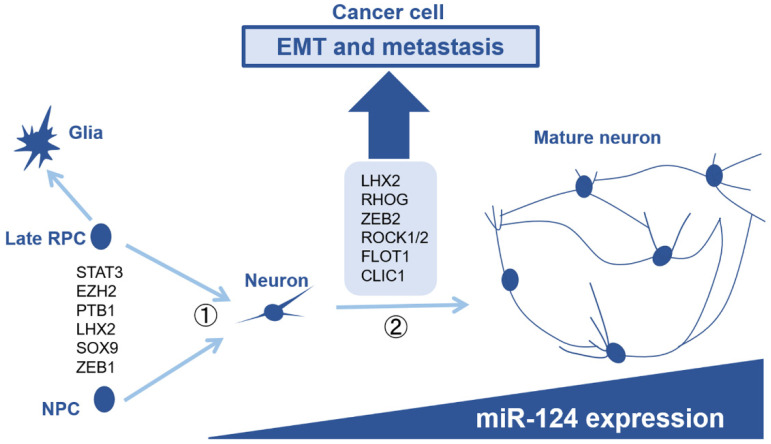
The role of microRNA-124 in neurogenesis and its tumor suppressive effect. miR-124 is strongly expressed in neurons, and its expression increases as the neurons mature. miR-124 is thought to act as a gatekeeper during the generation of neurons from non-neuronal cells, thus preventing the reversal of cell fate. In the process of neuronal maturation, miR-124 targets genes that contribute to proper neuronal migration, neurite formation, and synapse formation. Genes that contribute to neuronal maturation also contribute to EMT and metastasis in cancer cells. Abbreviations: RPC, retinal progenitor cell; NPC, neural progenitor cell; EMT, epithelial-mesenchymal transition.

Thus far, we have summarized the tumor-suppressive effects of miR-124 in cancer cells. However, miR-124 is strongly expressed in neurons typically and plays an important role in neuronal development. In addition, the expression level of miR-124 increases with neuronal maturation [9,118]. Therefore, it is thought that miR-124 plays various roles at each step in the development of the nervous system. During the development of the central nervous system, nascent neurons migrate to appropriate locations during maturation to form neurocircuits and stabilize them. In addition, neurons generally do not proliferate after their fate has been determined. Considering these features, many of the genes that have been identified as molecular mechanisms involved in miR-124-mediated tumor suppression are not specific to cancer cells but are also important in neuronal differentiation. Here, we linked the tumor suppressor function of miR-124 to actual neuronal development (Figure 3).

During brain development, neurons are derived from neural progenitor cells called radial glia. In contrast, in the retina, both neurons and glial cells are produced from common retinal progenitor cells. *STAT3* is an important target gene of miR-124 for its tumor suppressor effect, while in nervous system development, it is known to contribute to astrocyte differentiation [119]. In addition, P19 cells, an embryonic-derived teratoma cell line, can also differentiate into neurons and astrocytes. miR-124 reportedly targets *EZH2* to allow differentiation of P19 cells into neurons and suppresses their differentiation into astrocytes [120]. Therefore, miR-124 is also thought to play a role in suppressing glial cell differentiation. Overexpression of miR-124 in mouse retinal progenitor cells decreases the percentage of glial cell differentiation and increases the percentage of neuronal differentiation [121]. Thus, it is possible that *STAT3* and *EZH2* are also targeted in the active fate determination of neurons from retinal progenitor cells in actual in vivo conditions.

Furthermore, overexpression of miR-124 can induce the conversion of non-neuronal cells to neurons [122]. As well as *Ezh2* [123], among the target genes of miR-124 in cancer cell, polypyrimidine tract-binding protein 1 (*Ptbp1; PTB1* gene in humans) is known to be associated with such an effect [124]. PTBP1/PTB1 regulates cell type-specific alternative splicing. In neural stem cells, PTBP1/PTB1 functions to produce non-neuronal splicing isoform proteins. Suppression of *Ptbp1* by miR-124 results in the production of neural-specific isoforms in mice [125]. Such a difference in splicing isoforms is also known to affect the metabolism of cancer cells. Pyruvate kinase muscles (PKMs) have two splicing isoforms, the *PKM1* and *PKM2*. In cancer cells, especially colon cancer cells, the amount of PKM2 increases due to the regulation of splicing by *PTB1*, and PMK1 increases when *PTB1* expression is suppressed by miR-124. This alters the metabolic pathways of cancer cells and leads to a decrease in the Warburg effect of miR-124 [74]. Although it has not been found at the present time, it may be discovered in the future that PTB1-mediated metabolic control is also important for neuronal differentiation.

Newborn neurons then undergo a maturation process. Studies on miR-124-1 knockout mice have shown that miR-124 is essential for neuronal maturation [9]. miR-124 targets LIM-homeobox domain 2 (*Lhx2*), which is thought to regulate neuronal maturation [9]. The transcription factor LHX2 is expressed in neural stem cells and immature neurons, and is essential for neuronal production, maturation, and normal axonal projection [126,127,128]. Interestingly, in NSCLC, it is reported miR-124 targets *LHX2* and that its reduction is associated with inhibition of metastasis and invasion [64]. Furthermore, miR-124 reportedly targets *RHOG* and promotes dendritic branching during neuronal maturation [129]. In breast cancer, *RHOG* has been identified as a target gene of miR-124 in suppressing the metastasis of cancer cells [89]. Besides these, genes that contribute to neuronal maturation [130,131,132,133] appear to be linked to genes that contribute to malignant transformation, such as migration and invasion, in cancer cells [70,79,83,86,89,91,98] (Figure 3). Reportedly, zinc finger E-box binding homeobox 2 (*ZEB2*) is a target of miR-124 in breast cancer [98], and reduction of *ZEB2* suppresses EMT and metastasis. Although there are no clear examples regarding the important functional regulation of miR-124 to *ZEB2* in neurons, we believe that targeting of *ZEB2* by miR-124 is important in neuronal maturation as ZEB2 promotes axonal branching and regulates normal migration of interneurons [133].

## 7. Discussion

Each of the papers discussed in this review addresses several target genes as a mechanism for the anticancer effects of miR-124. However, since each study focuses on a unique gene, unidentified targets might be missed. We think transcriptome analysis is a necessary tool, although it may be difficult to find all of them, since repression of the target genes also occurs at the translational repression level. In addition, each single cancer cell may have different characteristics, even if they are from the same strain. Therefore, the diversity of the effects of miR-124 might be determined by analyzing it at the single-cell level. Furthermore, although there have been many reports on the anticancer effects of miR-124, it is thought that there are cancers for which miR-124 does not work. We believe that analysis of such cancers is also necessary for the application of miR-124 in the treatment of cancer.

Many experimental methods express miRNAs into cancer cells using gene transfer of pre-miRNAs by plasmid or viral vectors, and induction of these miRNAs by the Tet-ON system. For clinical methods, miRNAs themselves, or chemically modified miRNAs called miRNA mimics, packaged into nanoparticles and delivered to cancer cells for uptake may be a more realistic means of miRNA-based cancer therapy [134].

## 8. Conclusions

miR-124 showed anticancer effects in various stages of cancer progression, and was not only a replacement for existing treatment methods by itself, but also enhanced the effects of the existing treatment methods, probably due to the characteristic of miRNAs to regulate multiple target genes simultaneously.

In this review, we compared the anticancer effects of miR-124 with its effect on neural development. Despite the different functions of anticancer and neurogenic regulation, there are many common genes between the two. This means that neurodevelopmental functions may lead to new anticancer targets and anticancer effects may lead to new and important neurodevelopmental discoveries. Both fields can be made more progressive by paying attention to each other.

## Figures and Tables

**Table 1:** Anti-tumor function of microRNA-124 in glial lineage cancer.

TARGET GENES	EFFECTS	REF.
*AURKA*	Inhibition of cell proliferation and potentiation of the temozolomide TMZ-based chemosensitivity.	[45]
*CAPNS1*	Inhibition of cell migration and invasion.	[42]
*CDK4*	Conferring radio-sensitivity.	[51]
*CDK4*, *CDK6*, and *PPP1R13L*	Inhibition of cell migration, decrease in cell viability, and cell cycle arrest at the G0/G1 phase.	[47]
*CDK6*	Induce G1 cell cycle arrest.	[15]
*CDK6*	Decrease in cell proliferation and migration and conferring chemosensitivity to TMZ.	[48]
*CDK6*	Inhibition of cell proliferation.	[38]
*TEAD1*, *MAPK14*, and *SERP1*	Increase in cell death.	[41]
*EZH2*	Expression of miR-124/128/137 of artificial miRNA clusters, reduction of cell proliferation both in vitro and in vivo, and prolongation of survival in a mouse model.	[50]
*KITLG*, *NRP2*, *SEMA6D*, and *THBS1*	Regulation of self-renewal, apoptosis, and invasion.	[44]
*NRP1*	Arresting of cell cycle, inhibition of cell proliferation and migration, inhibition of tumor angiogenesis, and induction of apoptosis.	[49]
*PIM1*	Inhibition of cell proliferation, invasion, and aerobic glycolysis and promotion of apoptosis.	[43]
*PPP1R13L*	Inhibition of cell proliferation, G1/S transition, and invasiveness.	[40]
*SDCBP*	Decrease in malignancy of glioblastoma cells, inhibition of cell proliferation, migration, and invasion.	[46]
*SOS1*	Inhibition of cell proliferation.	[37]
*STAT3*	Reversing immunosuppression in the tumor microenvironment, enhancing T cell-mediated immune clearance, and inhibition of glioma growth.	[39]

TMZ: temozolomide.

**Table 2:** Anti-tumor function of miR-124 in lung cancer.

TARGET GENES	EFFECTS	REF.
*AKT2*	Arresting of cell cycle at the G0/G1 phase and inhibition of cell growth, colony formation, and tumor growth without apoptosis.Inhibition of migratory and invasive abilities.	[66]
*BECN1*, *RELA*, and *SQSTM1*	Disruption of autophagy and reduction of cell survival.	[59]
*CD164*	Inhibition of tumor cell proliferation, colony formation, migration, and induction of apoptosis.	[58]
*CDH2*	Inhibition of cell proliferation and invasion.	[68]
*EZH2*	Inhibition of tumor cell proliferation and inhibition of the EMT process.	[67]
*LHX2*	Attenuation of cellular migratory and invasive abilities.	[64]
*MYO10*	Inhibition of migration and metastatic ability.	[61]
*SNAI2*	Inhibition of invasion.	[56]
*STAT3*	Inhibition of cell proliferation and induction of apoptosis.	[55]
*STAT3*	Inhibition of cell growth and colony formation and induction of apoptosis.	[32]
*STAT3*	Inhibition of cell growth and colony formation and induction of apoptosis. Increasing radio sensitivity.	[63]
*STAT3*	Inhibition of invasion and metastasis capacities. Increasing sensitivity to cisplatin.	[65]
*TXNRD1*	Improving sensitization of radiation-resistant cells to radiation.	[62]
*ZEB1*	Inhibition of migration and invasion through suppressing EMT.	[57]

EMT: epithelial-mesenchymal transition.

**Table 3:** Anti-tumor function of miR-124 in colorectal cancer.

TARGET GENES	EFFECTS	REF.
*DDX6* and *PTB1*	Induction of apoptosis. Decreasing production of lactic acid, affecting the Warburg effect.	[74]
*VANGL1*, *MYH9*, and *SOX9*	Inhibition of tumorigenicity.	[69]
*PPP1R13L*	Inhibition of cell proliferation and tumor formation.	[71]
*PTB1*	Enhancement in oxidative stress and induction of apoptosis and autophagy.	[72]
*ROCK1*	Inhibition of cell proliferation, migration, and invasion.	[70]
*STAT3*	High-intensity focused ultrasound mediated inhibition of migration.	[75]
*STAT3*	Induction of apoptosis and inhibition of tumor growth.	[76]

**Table 4:** Anti-tumor function of miR-124 in liver cancer.

TARGET GENES	EFFECTS	REF.
*CASC3*	Inhibition of tumor growth.	[81]
*CDK6*, *IQGAP1*, *SMYD3*, and *VIM*	Inhibition of cell growth.	[77]
*CLIC1*	Inhibition of cell proliferation, migration and invasion.	[83]
*EZH2* and *ROCK2*	Inhibition of cell motility and invasion, and suppression of intrahepatic and pulmonary metastasis. Inhibition of EMT with impaired formation of stress fibers, filopodia, and lamellipodia.	[79]
*ITGAV* and *SP1*	Inhibition of migration and tumor metastasis.	[82]
*PIK3CA*	Cell cycle arrest at the G0/G1 phase.	[78]
*STAT3*	Inhibition of cell proliferation and induction of apoptosis.	[80]

EMT: epithelial-mesenchymal transition.

**Table 5:** Anti-tumor function of miR-124 in gastric cancer.

TARGET GENES	EFFECTS	REF.
*EZH2*	Inhibition of cell proliferation and colony formation and induction of apoptosis. Increased sensitization of 5-FU.	[87]
*EZH2* and *JAG1*	Inhibition of cell growth, migration, invasion, and tumor growth.	[88]
*ROCK1*	Inhibition of cell proliferation, migration, and invasion.	[86]
*SPHK1*	Inhibition of cell proliferation and tumorigenicity.	[84]
*SPHK1*	Suppression of cell proliferation and invasion.	[85]

5-FU: 5-fluorouracil.

**Table 6:** Anti-tumor function of miR-124 in breast cancer.

TARGET GENES	EFFECTS	REF.
*AKT2*	Inhibition of cell proliferation, migration, and invasion.	[94]
*CBL*	Inhibition of cell proliferation and invasion.	[95]
*CD151*	Inhibition of proliferation via cell cycle arrest but does not induce apoptosis. Reduction of invasive and metastatic potential.	[90]
*CDK4*	Inhibition of cell proliferation.	[93]
*FLOT1*	Inhibition of cell growth and migration.	[91]
*IL11*	Inhibition of the survival and differentiation of osteoclast progenitor cells through cancer cell-derived microRNA-124.	[96]
*CTGF*, *ITGB1*, *RHOG*, and *ROCK1*	Inhibition of metastasis.	[89]
*SNAI2*	Inhibition of cell colony formation and pulmonary metastasis.	[92]
*STAT3*	Inhibition of cell proliferation and invasion.	[97]
*STAT3*	Improving sensitization of doxorubicin.	[100]
*ZEB2*	Inhibition of cell growth and migration and EMT.	[98]

EMT: epithelial-mesenchymal transition.
